# Campbell Collaboration: Reflection on growth and cultivation from 2017 to 2021

**DOI:** 10.1002/cl2.1204

**Published:** 2021-12-17

**Authors:** Vivian A. Welch

In 2021, *Campbell Systematic Reviews* has continued to grow and diversify in its coverage of social science content areas, while maintaining its productivity in publishing high quality, policy‐relevant reviews and evidence and gap maps. In reflecting on this past year, I am reminded of my vegetable and fruit gardens, which I expanded during increased time at home during the COVID‐19 lockdowns over the past two summers.

In terms of growth, the Campbell Board approved a new Coordinating Group on Child Welfare and a new Sub‐Group of the Social Welfare Coordinating Group, Sexual Orientation and Gender Identity. Our Methods Coordinating Group defined seven types of empirical methods research which it considers, including innovative methods papers, systematic reviews and guidance (Aloe & Garside, 2021). The first methods article of a joint series on evidence synthesis technology with the Collaboration on Environmental Evidence was published in November 2020 (Haddaway et al., 2020).

In terms of quality, our baseline assessment of Campbell reviews found that the majority of Campbell reviews are high or moderate quality, and their quality has increased over time (Wang et al., 2021). As a member of the Committee On Publication Ethics (COPE), all of our editors complete training on the use of COPE editorial flowcharts and participate in regular editors' training on hot topics in peer review and editorial processes. We are currently creating online training modules for prospective authors, in collaboration with Carnegie Melon University.

Our editorial board has prioritized five areas for the next year: (1) enabling public comments and debate for all of our articles, (2) developing a policy on equity, diversity and inclusion for our editorial teams and our content, (3) updating our conflict of interest policy, (4) screening for adherence to methodological expectations, and (5) adopting a transparent peer review policy. For each of these, as an evidence‐based organization, we will monitor implementation and assess effects on production timeliness and quality.

We have built on the enthusiasm of members of the Campbell community, with a number of projects underway led by our members. These include, but are not limited to, the development of a Campbell Wikipedia Project (Campbell Collaboration Wikipedia Project, 2021), working groups to develop guidance for qualitative evidence synthesis, guidance on risk of bias appraisal for nonrandomized studies and guidance on information specialist peer review of search strategies.

As a measure of our impact, in May 2021, *Campbell Systematic Reviews* was accepted by Clarivate in the Emerging Sources Citation Index, which is a big step forward on our pathway to seeking a journal impact factor. We have also almost doubled the number of stories of policy influence in the last year. We track these influence stories using Altmetric, available on all of our articles since January 2019. Campbell systematic reviews have influenced international guideline development, intervention development and implementation, and informed policy discussions and research agendas.

The ongoing COVID‐19 pandemic and its social and economic consequences require evidence to inform policy both now and in the post‐COVID recovery in all of the areas covered by *Campbell Systematic Reviews*. This evidence‐base needs to be transparent, rigorous and comprehensive. It needs to identify not only the policy implications, but also the uncertainties in the evidence‐base which require further research. Some recent examples are reviews on homelessness (Keenan et al., 2021), nutrition (Lassi et al., 2021), school‐based interventions for students at risk of academic difficulties (Dietrichson et al., 2021), risk factors for radicalization (Wolfowicz et al., 2021), using big data for international development (Rathinam et al., 2021), employment for persons with autism spectrum disorders (Fong et al., 2021), and multisystemic family therapy (Littell et al., 2021).

The Campbell Collaboration is at an exciting crossroads. We are currently exploring transition to a new host institution to codevelop our long‐term sustainability as a member‐driven, open‐access organization which is committed to providing the best evidence to inform social policy decisions. This will be the fifth change in host organization of Campbell, and is happening at a time when we are at our strongest in history in terms of production, impact and membership, thanks to the current leadership.

Returning to my gardens, I plan my crops and planting locations based on evidence about complementary crops to minimize pests, soil composition, spacing, orientation, sun exposure, fertilizer and water to maximize production. Despite this planning, some crops under‐delivered due to unforeseen pest invasions or weather extremes. Others outperformed expectations. Monitoring of these variations in outcome, and exploring possible causes gives insight into how to plan in future years.

At Campbell, we welcome your submissions of high quality evidence synthesis and methodological research focused on evidence synthesis. As with my gardens, we will continue to monitor our growth and influence so we can make any needed corrections to achieve our vision of better evidence for a better world.
